# Post-training depletions of basolateral amygdala serotonin fail to disrupt discrimination, retention, or reversal learning

**DOI:** 10.3389/fnins.2015.00155

**Published:** 2015-05-11

**Authors:** Jesus G. Ochoa, Alexandra Stolyarova, Amandeep Kaur, Evan E. Hart, Amador Bugarin, Alicia Izquierdo

**Affiliations:** ^1^Department of Psychology, University of California, Los AngelesLos Angeles, CA, USA; ^2^Brain Research Institute, University of California, Los AngelesLos Angeles, CA, USA

**Keywords:** cognitive flexibility, reward learning, serotonin, amygdala, retention, 5, 7-DHT, 5-HT

## Abstract

In goal-directed pursuits, the basolateral amygdala (BLA) is critical in learning about changes in the value of rewards. BLA-lesioned rats show enhanced reversal learning, a task employed to measure the flexibility of response to changes in reward. Similarly, there is a trend for enhanced discrimination learning, suggesting that BLA may modulate formation of stimulus-reward associations. There is a parallel literature on the importance of serotonin (5HT) in new stimulus-reward and reversal learning. Recent postulations implicate 5HT in learning from punishment. Whereas, dopaminergic involvement is critical in behavioral activation and reinforcement, 5HT may be most critical for aversive processing and behavioral inhibition, complementary cognitive processes. Given these findings, a 5HT-mediated mechanism in BLA may mediate the facilitated learning observed previously. The present study investigated the effects of selective 5HT lesions in BLA using 5,7-dihydroxytryptamine (5,7-DHT) vs. infusions of saline (Sham) on discrimination, retention, and deterministic reversal learning. Rats were required to reach an 85% correct pairwise discrimination and single reversal criterion prior to surgery. Postoperatively, rats were then tested on the (1) retention of the pretreatment discrimination pair, (2) discrimination of a novel pair, and (3) reversal learning performance. We found statistically comparable preoperative learning rates between groups, intact postoperative retention, and unaltered novel discrimination and reversal learning in 5,7-DHT rats. These findings suggest that 5HT in BLA is not required for formation and flexible adjustment of new stimulus-reward associations when the strategy to efficiently solve the task has already been learned. Given the complementary role of orbitofrontal cortex in reward learning and its interconnectivity with BLA, these findings add to the list of dissociable mechanisms for BLA and orbitofrontal cortex in reward learning.

## Introduction

Control over inappropriate responding plays a pivotal role in adaptive decision making. Poor inhibitory control is a characteristic of a wide range of psychiatric disorders, including obsessive-compulsive disorder (Chamberlain et al., 2005), attention-deficit-hyperactivity disorder (Itami and Uno, 2002), addiction (Brewer and Potenza, 2008; Winstanley et al., 2010), and personality disorders (Soloff et al., 2003; Lieb et al., 2004). Reversal learning, measuring the ability to actively suppress prepotent responding, is a broadly-used assay of flexible reward learning and has been proposed as an index for some psychopathology (Izquierdo and Jentsch, 2012).

The literature on the modulation of cognitive flexibility by serotonin (5-HT) is vast. Substantial evidence from studies employing pharmacological manipulations of serotonergic neurotransmission by receptor antagonist administration (Boulougouris and Robbins, 2010), selective toxin-mediated depletions of 5-HT and destruction of 5-HT terminals (Clarke et al., 2004, 2007; Masaki et al., 2006) combined with genetic studies (Homberg et al., 2007; Brigman et al., 2010; Jedema et al., 2010) have established a prominent role for this neurotransmitter in reversal learning performance in different species. Overall, these studies suggest that global reductions of serotonin levels are associated with a higher degree of perseveration and poor response control.

Among other brain nuclei, the serotonergic system innervates prefrontal cortex (PFC), basolateral amygdala (BLA), and nucleus accumbens (Kapur and Remington, 1996; McQuade and Sharp, 1997), regions critical for flexible reward learning (Cools et al., 2002; Ghahremani et al., 2010; Izquierdo and Jentsch, 2012). Several lines of evidence point to a selective role for 5-HT within subregions of the PFC in the modulation of behavioral flexibility (Dalley et al., 2002; Clarke et al., 2004; Winstanley et al., 2006). The role of 5-HT in BLA in reward learning is not as well understood. The relative paucity of systematic examination is surprising given that the lateral amygdala receive dense serotonergic inputs from the dorsal raphé (Sadikot and Parent, 1990) and expresses several subtypes of serotonergic receptors (Xu and Pandey, 2000; Mascagni and McDonald, 2007). The activity levels of BLA neurons, the degree of inhibition, and synaptic responsiveness are modulated by 5-HT signaling (Rainnie, 1999; Yamamoto et al., 2012), suggesting an important role of this neurotransmitter in BLA function.

BLA has been implicated in the performance of tasks measuring cognitive flexibility, including reversal learning, although the results are contradictory with some studies reporting normalization (Stalnaker et al., 2007), enhancement (Izquierdo et al., 2013), or deterioration of performance (Churchwell et al., 2009) following manipulations. Given that reversal learning is a net manifestation of multiple processes, including inhibition of a previously learned association, sensitivity to reward feedback following choice (Stolyarova et al., 2014), degree of perseveration, and learning of new stimulus-outcome contingencies (Roberts, 2006; Izquierdo and Jentsch, 2012), the lack of agreement in these results likely hinge on the particular demands of the task, animals' motivational state, the order of the task presentation (pre- or post-training manipulations), or the specificity of the manipulation. Irrespective of the methodological differences, it appears that BLA is selectively important in updating responses to changes in reward value (Coleman-Mesches et al., 1996; Baxter and Murray, 2002; Liao and Chuang, 2003; Belova et al., 2008) and sensitivity to negative feedback (Rudebeck and Murray, 2008; Izquierdo et al., 2013). Rather than integrating the information across time, the BLA-lesioned animals appear to be guided by immediate outcomes, potentially leading to an enhanced win-stay/lose-shift strategy. Thus, an effect on reversal learning can be expected under some but not all experimental protocols.

A specific role for 5-HT in amygdala in reversal learning has previously been suggested (Masaki et al., 2006). In this study the levels of 5-HT in amygdala were negatively correlated with the number of sessions required by the animals to advance to both discrimination and reversal criterion on a go/no-go task. Similarly, Izquierdo et al. (2012) reported impaired stimulus-reward association learning despite intact motivation after systemic depletions of 5-HT after parachlorophenylalanine, a tryptophan hydroxylase (TPH) inhibitor. However, both of those studies employed systemic pharmacological manipulations, which also produced significant 5-HT reductions in OFC, mPFC, and hippocampus, among the brain regions examined. Therefore, the reversal learning effect reported in Masaki et al. (2006) cannot be attributed exclusively to amygdalar 5-HT depletions. To our knowledge, only one experimental study has been conducted thus far to suggest a causal role for 5-HT in BLA in mediating reversal learning. Rygula et al. (2014) observed impaired probabilistic reversal learning performance in marmosets following 5-HT depletion of amygdala. The impairment resulted from increased effectiveness of misleading feedback and decreased overall reinforcer sensitivity. However, it is not known whether 5-HT neurotransmission within BLA is necessary for deterministic, non-probabilistic two-choice reversal learning: wherein one stimulus is rewarded 100% of trials and the other stimulus rewarded 0% of trials.

To test whether the BLA-specific depletion of 5-HT produces impairments in reversal learning, we first assessed animals' performance on initial pairwise discrimination and reversal learning, then performed selective 5-HT depletions within this region to examine their subsequent performance on retention of preoperative reward contingencies, novel pairwise discrimination, and reversal learning.

## Experimental procedures

### Subjects

Fifteen experimentally naïve male Long-Evans (Charles Rivers Laboratories, Hollister, CA) rats (PND 50, weighing between 280 and 300 g at the beginning of the study) were pair housed in rooms with automatically regulated lighting (12 h light/dark cycle; lights on at 06:00), maintained on rat chow (Rodent Lab Chow 50#) and water *ad libitum* until training commenced. Upon arrival, the rats were allowed to habituate for 3 days prior to being handled for 5 days (10 min per rat). Following handling, the rats were food restricted to no less than 85% of their free-feeding body weight to ensure motivation to work for sucrose pellets (Bio-Serv, Frenchtown, NJ) in the operant chambers while water was available *ad libitum*. Body weights were monitored at least 3 times per week. Behavioral testing took place between 08:00 and 16:00 h during the rats' inactive period, consistent with previous studies in our lab.

### Behavioral apparatus

Behavioral testing was done in eight operant conditioning chambers (Model 80604 Lafayette Instrument Co., Lafayette, IN) that were housed within sound- and light- attenuating cubicles. Each chamber was equipped with a house light, tone generator, video camera, and LCD touchscreen opposing the pellet dispenser. The pellet dispenser delivered single 45-mg dustless precision sucrose pellets. Software (ABET II TOUCH) controlled touchscreen stimuli presentation, tone generation, tray- and house-light illumination, and pellet dispensation.

### Behavioral pre-training

**Figure 1:**
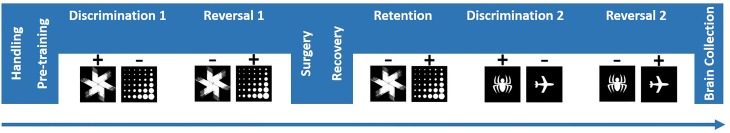
**Order of training, testing, and surgical procedures**. Following a series of pre-training phases rats were presented with two novel stimuli with predetermined reinforcement contingencies. The software enabled either a reward event as a result of nosepoking the correct stimulus, or a punishment as a result of nose poking the incorrect stimulus; the latter consisting of a 5 s “time out” wherein rats were unable to initiate the next trial. Criterion for advancement was 60 correct nose pokes at 85% correct responses to the stimulus within 45 min, on each of two consecutive days. Upon reaching criterion on this phase, the rats were tested on a reversal of the reward contingencies. Once the reversal learning criterion was met rats received bilateral injections of 5,7-DHT in the BLA (Depletion) or saline (Sham). Following a 7-day recovery period, rats were tested for retention of the previously learned reversal task as well as new discrimination and reversal learning problems.

The order of training, testing, and surgical procedures is outlined in Figure 1. The pre-training protocol, adapted from Kosheleff et al. (2012) and Izquierdo et al. (2010), consisted of a series of phases: Habituation, Initial Touch Training (ITT), Must Touch Training (MTT), Must Initiate Training (MIT), and Punish Incorrect Training (PIT) designed to train rats to nose-poke, initiate a trial, and discriminate between stimuli. During habituation, rats were required to eat five pellets out of the pellet dispenser inside of the chambers within 15 min before exposure to any stimuli on the touchscreen. ITI began with the display of white graphic stimuli on the black background of the touchscreen. During this stage a trial could be terminated for one of two reasons: if a rat touched the displayed image, or if the image display time (40 s) ended, after which the stimulus was removed and black background displayed. The disappearance of the image was paired with the onset of a “reinforcer event”: dispensation of one (image time ended) or three (image touched) sucrose pellets, a 1 s tone, and an illumination of the tray-light. Trials were separated by a 10 s ITI. In MTT, a trial could be terminated only if the rat touched the image, which then disappeared followed by reward delivery. Following successful acquisition of stimulus to reward relationship the rat had to learn to initiate a trial by nosepoking and exiting the reward magazine (MIT). Magazine entry was accompanied by auditory feedback. For all the stages, the criterion for advancement into the next stage was set to 60 rewards consumed in 45 min. During the last stage of pre-training rats were exposed to punishment (i.e., “time out” time during which a new trial could not be initiated) upon an incorrect response (PIT). The criterion for PIT was set to 60 rewards consumed in 45 min across two consecutive days.

### Pre-operative behavioral testing

The animals were given one testing session per day until the criterion was reached and were restricted to a maximum of 60 correct responses per testing session. Rats were presented with two novel, white, equiluminescent stimuli that differed only in shape with predetermined reinforcement contingencies. The software enabled either a reward event in the form of sugar pellet dispensation, paired with house-light illumination and auditory feedback, as a result of nose poking the correct stimulus, or a punishment as a result of nose poking the incorrect stimulus; the latter consisting of a 5 s “time out” wherein rats were unable to initiate the next trial. Trials were separated by a 5 s ITI. If the rat committed an error and received a punishment, a correction trial was administered to prevent side bias formation: this consisted of the same spatial (left/right) presentation of the stimulus until the rat nose poked correctly. Spatial configuration of stimuli presentation occurred pseudo randomly, the stimulus could not have appeared on the same side of the screen more than three times in a row except during correction trial. Stimulus assignment was counterbalanced across treatment groups. Criterion for advancement was 60 correct nose pokes at 85% correct responses to the stimulus within 45 min, on each of two consecutive days. Upon reaching criterion on this phase, the rats were tested on a reversal of the reward contingencies. Parameters for the reversal phase were identical to the visual discrimination learning phase, with the exception that the reward contingencies were reversed.

### Surgery

Rats were treated with Desipramine (10 mg/kg) 30 min before surgery, anesthetized with isoflurane (2–2.5%, to effect) through a nosecone and mounted on a stereotaxic apparatus (Model #963, Kopf Instruments, Tujunga CA). Respiratory rate and body temperature were monitored throughout the surgery. The skin was incised (anterior to posterior), retracted using hemostats, and the head position was adjusted to fit Bregma and Lambda on the same horizontal plane. Over the target area, small burr holes (2 mm diameter) were drilled bilaterally on the skull for the placement of an injection needle. A 10 μL Hamilton syringe was mounted and placed on an infusion pump and connected to an injection needle with polyethelyne tubing. Rats received two infusions per hemisphere (0.1 and 0.2 μL per site) of 5,7-DHT (5,7-dihydroxytryptamine, 20 mg/mL) bilaterally in the BLA (Depletion, *n* = 8) and sham-operated animals (Sham, *n* = 7) received 0.9% saline in the same sites. The total dose of 5,7-DHT administered was 6 μg per hemisphere or 12 μg per animal, which falls within the range of doses previously reported to produce specific and reliable 5-HT depletions associated with behavioral effects (Sommer et al., 2001; Macedo et al., 2002; Izumi et al., 2012; West et al., 2013).

The coordinates used for the injections were adapted from a previous report (Burke et al., 2007) were as follows: Site 1 (0.2 μL) AP = +2.8 mm; ML, ±5.0 mm; DV = −8.4 mm; Site 2 (0.1 μL) AP = +2.8 mm; ML, ±5.0 mm; DV = −8.1 mm from bregma. After the last infusion, the incision was sutured and warmed sterile saline (1 mL, s.c.) was administered. The rats were placed on a heating pad and kept in recovery until ambulatory before being put back into the vivarium.

### Post-operative behavioral testing

Following a 7-day recovery period, rats were put back on food restriction and tested for retention of the previously learned reversal task, using procedures identical to pre-operative reversal learning testing. Criterion for advancement was 60 correct nose pokes at 85% correct responses to the stimulus within 45 min, on each of two consecutive days. Upon completion of the retention stage, two novel stimuli were presented in a new discrimination and reversal phase.

### Immunohistochemistry

Depletions were verified using immunohistochemistry for the marker TPH. Following behavioral testing, rats were humanely euthanized with an overdose of sodium pentobarbital and perfused transcardially with 0.9% Saline buffer, followed by a 10% formaldehyde. The brains were extracted and post-fixed in 10% formaldehyde for 24 h, then transferred into a 30% sucrose solution until the brain sank to the bottom of the 5 ml scintillation vial. Thirty five micrometer coronal sections were cut on a cryostat (−20°C) and placed in 0.9% saline. Sections were rinsed in 0.9% saline for 5 min, permeabilized for 90 min at room temperature on an agitator in 0.3% Triton-X, 3% Normal Goat Serum, and 0.9% saline. After blocking, sections were washed 3 times for 5 min in 0.9% saline. Primary incubation period at 4°C on an agitator lasted for 72 h in a 1:500 rabbit anti- TPH, 0.3% Triton-X, 3% Normal Goat Serum, and PBS solution. Sections were then washed 3 times for 5 min in 0.9% saline. Secondary antibody was incubated in low light at room temperature for 3 h in a 1:400 goat-anti-rabbit FITC in 0.3% Triton-X, 3S% Normal Goat Serum in PBS. Sections were then washed 3 times for 5 min in 0.9% saline. After the slides were allowed to dry completely, they were cover slipped with 100 μL of Ultra Cruz. Zeiss Axio Observer Z1 was used for visualization and software Slidebook 5.5 for quantification of the staining.

### Statistical analyses

Software package SPSS (SAS Institute, Inc., Version 16.0) was used for statistical analyses. Statistical significance was noted when *p*-values were less than 0.05, and a trend toward significance was noted when *p*-values were 0.05–0.06. Shapiro Wilk tests of normality, Levene's tests of equality of error variances, Box's tests of equality of covariance matrices, and Mauchly's tests of sphericity were used to characterize the data structure. The learning data were analyzed with omnibus repeated-measure ANOVAs (rmANOVAs). Three parameters were considered in learning analyses: sessions to criterion, total number of committed errors, and performance accuracy (i.e., percent correct) across sessions for each testing phase. Sessions to criterion and total errors on discrimination and reversal learning phases were subjected to rmANOVA with time (pre- and post-operatively) as within- and treatment group (Depletion vs. Sham) as between-subject factors. Session performance accuracy data were analyzed with omnibus rmANOVA with time (pre- and post-operatively) and session as within- and treatment group as between-subject factors. Retention data were analyzed with independent samples *t*-tests (sessions to criterion and total error), and rmANOVA with session as within- and treatment group (Depletion vs. Sham) as between-subject factors (session percent correct). Immunohistochemistry data were analyzed with ANOVA with hemisphere (left vs. right) as within- and treatment group (Depletion vs. Sham) as between-subject factors. Where the assumptions of sphericity were violated, Greenhouse-Geisser *p*-value corrections were applied (Epsilon < 0.75).

## Results

### Immunhistochemical verification of 5,7-DHT lesions

**Figure 2:**
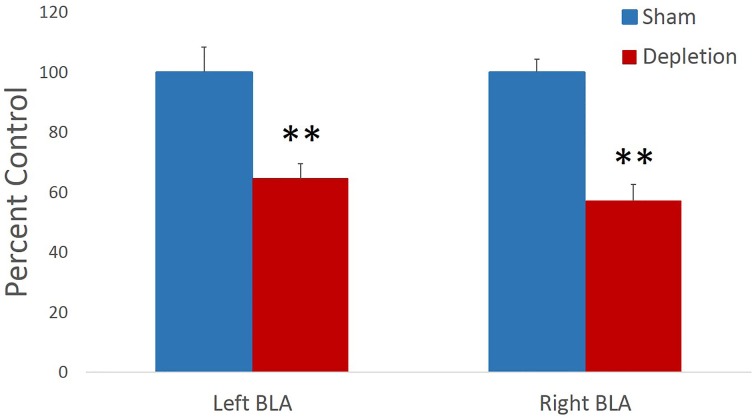
**5,7-DHT infusions produced a long-lasting moderate 5-HT depletion in BLA**. Following behavioral testing (mean number of days after the surgery = 68), TPH levels were significantly reduced in depletion compared to control group and not different between the left and right hemispheres.

5,7-DHT infusions produced a reliable moderate 5-HT depletion in BLA. Immunohistochemistry data were analyzed with ANOVA with hemisphere (left vs. right) as within- and treatment group (Depletion vs. Sham) as between-subject factors. Following the behavioral testing (mean number of days after the surgery = 68), the TPH levels were significantly reduced in depletion compared to control group: mean effect of treatment group [*F*_(1, 12)_ = 46.395, *p* < 0.001 Figure 2]; and not different between the left and right hemispheres [*F*_(1, 12)_ = 0.367, *p* = 0.556]. The mean levels of 5-HT depletion by the end of behavioral testing were 64.53% for the left and 51.01% for the right hemispheres. Individual TPH staining data are presented in Table 1.

**Table 1:** **Individual immunohistochemical data**.

**Animal**	**Percent control**		**Days post-surgery**
	**Left**	**Right**	
**SHAM**			
2Q	67.04	116.90	59
4Q	81.18	95.03	57
9Q	113.08	90.32	78
11Q	116.99	89.16	77
12Q	110.54	105.83	77
15Q	111.18	102.76	76
Average	100	100	70.67
**DEPLETION**			
1Q	65.59	52.57	59
3Q	81.91	56.14	58
5Q	59.85	52.34	57
6Q	63.64	69.52	55
10Q	87.96	56.86	77
13Q	50.09	56.68	76
14Q	58.27	23.98	76
16Q	48.91	39.99	76
Average	64.53	51.01	66.75

*Depletions in the left and right hemisphere were verified using immunohistochemistry for the marker tryptophan hydroxylase (TPH)*.

### 5-HT-depleted animals had intact memory for previously learned task contingencies

**Figure 3:**
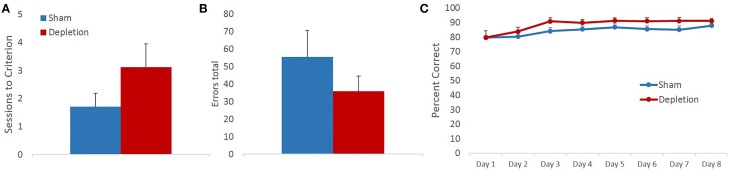
**5-HT-depleted animals had intact memory for previously learned task contingencies**. Memory for the previously learned task contingencies was assessed by retention of the pre-operative reversal task 7 days after the surgery. There was no statistical difference between treatment groups on **(A)** sessions to criterion, or **(B)** total errors. **(C)** All animals improved their performance with time. 5-HT depletions in BLA had no effect on performance accuracy on any of the sessions of retention task.

Memory for previously learned task contingencies was assessed by retention of the pre-operative reversal task 7 days after the surgery. Sessions to criterion and total number of errors on a retention task were analyzed with independent sample *t*-tests. There was no statistical difference between treatment groups on either of the measures [sessions to criterion *t*_(13)_ = 1.414, *p* = 0.181, Figure 3A; total errors *t*_(13)_ = −1.159, *p* = 0.267 Figure 3B]. Group differences in performance accuracy on each testing day were further analyzed with rmANOVA with session as within- and treatment group (Depletion vs. Sham) as between-subject factors. All animals improved their performance with time: main effect of testing session [*F*_(7,91)_ = 5.328, *p* = 0.016]. 5-HT depletions of BLA had no effect on performance accuracy on any of the sessions of retention task (Figure 3C): no main effect of group [*F*_(1, 13)_ = 0.017, *p* = 0.899] or session × group interaction [*F*_(7,91)_ = 0.255, *p* = 0.744].

### 5-HT depletions of BLA do not affect acquisition of visual discrimination learning task

**Figure 4:**
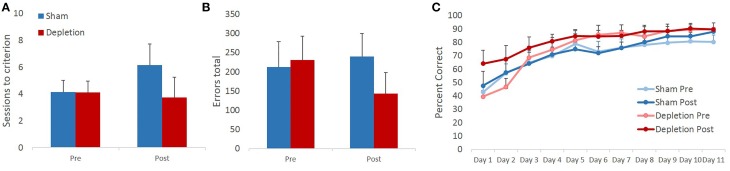
**5-HT depletions in BLA do not affect acquisition of a visual discrimination learning task. (A)** 5-HT depletions did not impair animals' ability to acquire the discrimination task. We anticipated faster acquisition of a second novel discrimination as a result of practice with the task. However, no intraproblem transfer of learning was observed. **(B)** Similarly, no practice effect was observed on total number of errors. There were also no treatment group differences in the total number of committed errors. **(C)** All animals improved their performance with time. 5-HT depletion did not affect animals' learning rate.

Sessions to criterion and total errors made on the pairwise visual discrimination learning task were analyzed with rmANOVA with time (pre- and post-operatively) as within- and treatment group (Depletion vs. Sham) as between-subject factors. The analyses revealed that 5-HT depletions did not impair animals' ability to acquire the discrimination task: no main effects of treatment group [*F*_(1, 13)_ = 0.952, *p* = 0.347] or time × treatment group interaction [*F*_(1, 13)_ = 0.889, *p* = 0.363] on sessions to criterion were observed. We anticipated faster acquisition of the second novel discrimination as a result of practice with the task. However, rmANOVA revealed no main effect of time [*F*_(1, 13)_ = 0.416, *p* = 0.53] on sessions to criterion (Figure 4A). Similarly, no practice effect was observed on total number of errors [*F*_(1, 13)_ = 0.274, *p* = 0.61]. There were also no treatment group differences [main effect *F*_(1, 13)_ = 0.376, *p* = 0.551; interaction *F*_(1, 13)_ = 1.009, *p* = 0.334] in the total number of committed errors (Figure 4B).

Some experimental manipulations are known to produce a stage-dependent effect on learning, in which case facilitated performance at a later stage may mask the impairment observed early in learning. Therefore, animals' performance was analyzed on a session-by-session basis. Performance accuracy was analyzed with rmANOVA with time (pre- and post-operatively) and session as within- and treatment group (Depletion vs. Sham) as between-subject factors. All animals improved their performance with time as evidenced by a highly significant main effect of testing session [*F*_(10,130)_ = 27.493, *p* < 0.0001]. Similarly to sessions to criterion and total errors data, the analyses revealed no improvement due to practice effect on any of the testing days: no main effect of time [*F*_(1, 13)_ = 0.738, *p* = 0.406] or time × session interaction [*F*_(10,130)_ = 1.186, *p* = 0.32] were observed. 5-HT depletion did not affect animals' learning rate: no main effect of group [*F*_(1, 13)_ = 1.705, *p* = 0.214], time × group [*F*_(1, 13)_ = 0.219, *p* = 0.647], session × group [*F*_(10,130)_ = 0.416, *p* = 0.704] or time × session × group interaction [*F*_(10,130)_ = 1.07, *p* = 0.355] (Figure 4C).

### 5-HT-depleted animals adapted their responses following a change in reward contingencies at a rate comparable to controls

**Figure 5:**
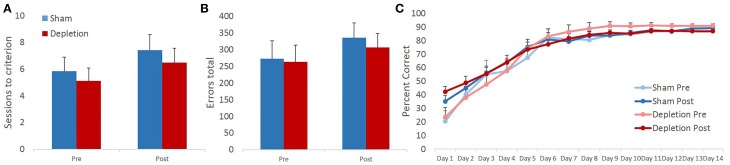
**5-HT-depleted animals switched their responses following the change in stimulus-outcome contingencies at a rate comparable to controls**. Similar to pairwise discrimination learning, 5HT-depleted were indistinguishable from sham-operated animals on all of the measures. **(A)** In contrast to discrimination learning, in reversal all animals required more sessions to reach criterion post- compared to pre-surgery. **(B)** No significant effects were observed for the total number of committed errors during the reversal phase. **(C)** There was a robust main effect of session indicating that all animals improved their performance with repeated testing. 5-HT depletion of BLA had no effect on animals' reversal learning performance.

Sessions to criterion and total errors made on the reversal learning task were analyzed with rmANOVA with time (pre- and post-operatively) as within- and treatment group (Depletion vs. Sham) as between-subject factors. Similarly to the pairwise discrimination learning, 5HT-depleted group was indistinguishable from sham-operated animals on either of the measures. rmANOVA revealed no main effect of group [*F*_(1, 13)_ = 0.346, *p* = 0.567] or time × group interaction [*F*_(1, 13)_ = 0.043, *p* = 0.839]. In contrast to discrimination learning where there was no effect of time on any of the measures, rmANOVA detected significant main effect of time for sessions to criterion on reversal learning [*F*_(1, 13)_ = 9.667, *p* = 0.008]. Animals' performance changed in a direction opposite of the anticipated, all animals required more sessions to reach reversal learning criterion post- compared to pre-operative (Figure 5A). No significant effects were observed for total number of committed errors during the reversal phase: no main effect of time [*F*_(1, 13)_ = 1.866, *p* = 0.195], group [*F*_(1, 13)_ = 0.123, *p* = 0.731], or time × group interaction [*F*_(1, 13)_ = 0.067, *p* = 0.8] (Figure 5B).

To probe for between-group differences in learning rates during the reversal phase of the task, session-by-session performance accuracy data were analyzed with omnibus rmANOVA with time (pre- and post-operatively) and session as within- and treatment group (Depletion vs. Sham) as between-subject factors. There was a robust main effect of session indicating that all animals improved their performance with repeated testing [*F*_(13,169)_ = 78.849, *p* < 0.0001]. Minimal effects of pre-operative testing on subsequent reversal learning were observed: no main effect of time [*F*_(1, 13)_ = 0.42, *p* = 0.528], but a trend for time × session interaction [*F*_(13,169)_ = 2.449, *p* = 0.067]. *Post-hoc* analyses further revealed a significant difference on day 1 of reversal learning between pre- and post-operative assessments with rats obtaining higher early accuracy levels during post-operative testing [*t*_(14)_ = −2.447; *p* = 0.027]. 5-HT depletion of BLA had no effect on animals' reversal learning performance. There was no main effect of group [*F*_(1, 13)_ = 0.22, *p* = 0.647], group × time [*F*_(1, 13)_ = 0.382, *p* = 0.547], group × session [*F*_(13,169)_ = 0.348, *p* = 0.746], or group × time × session [*F*_(13,169)_ = 0.44, *p* = 0.755] interactions (Figure 5C).

## Discussion

The results of the present investigation revealed intact discrimination and reversal learning following 5,7-DHT-mediated serotonergic depletions of BLA. The lesioned animals demonstrated intact memory for previously learned associations as evidenced by lack of between group differences on the retention task. These animals were unimpaired relative to controls at acquiring a novel discrimination task and flexibly adapted their responses following a change in reward contingencies, at a rate comparable to Sham animals.

The lack of group differences in retention of a previously learned stimulus-reward associations and acquisition of a novel discrimination learning problem is in line with previous observations demonstrating that amydgala lesions or inactivations have no effect on the memory for stimulus-reward associations (Izquierdo and Murray, 2007; Stalnaker et al., 2007; Izquierdo et al., 2013). It further strengthens the notion that BLA is not required in situations when the associations are stable, but instead involved in updating reward values following a change in stimulus-reward assignment.

Whereas, the lack of a stimulus-reward memory effect was expected, the lack of effect on the reversal phase of the experiment was surprising, given the prominent role of 5-HT in modulation of BLA activity and function (Rainnie, 1999; Mascagni and McDonald, 2007; Yamamoto et al., 2012), and previous results demonstrating the involvement of this brain region in cognitive flexibility (Stalnaker et al., 2007; Churchwell et al., 2009; Izquierdo et al., 2013). The mean levels of 5-HT depletion by the end of behavioral testing were 35.47% for the left and 48.99% for the right hemispheres. However, brain tissue was collected on average 68 days after the surgery, and more substantial reductions in 5-HT levels are expected at earlier time points when the behavioral assessment took place. Notably, these depletion levels are comparable to those reported in previous study where attenuated probabilistic reversal learning performance was observed (Rygula et al., 2014). The most compelling evidence for a causal role of 5-HT BLA depletions in reversal learning impairment comes from the aforementioned study by Rygula et al. (2014) that employed a probabilistic learning task. Although the explanation and analysis of the behavior presented by the authors suggesting a direct role for 5-HT in BLA in reward learning and reversal is plausible, another possibility exists. It is well established that BLA structural and functional integrity is critical for appropriate responses to reward devaluation. For example, animals with BLA-OFC disconnections are not able to update their response strategy in the face of changing reward value regardless of whether they need to rely on stored representations of reward value (i.e., during extinction) or if the devalued reward is delivered (Zeeb and Winstanley, 2013). Although not analyzed by Rygula et al. in such a manner, risk is itself a strong discounting parameter, factoring into reward valuation along with delay and effort demands. The previous studies implicating BLA in reward devaluation have suggested decreased sensitivity to probability costs. Patients with damage to amygdala frequently make disadvantageous, risky choices (Bechara et al., 1999; Brand et al., 2007), especially in tasks stressing potential gains (Weller et al., 2007). The difference in risk or uncertainty cost associated with each response options in the Rygula et al. (2014) study is the change from the initial outcome probability of 80:20 and its reversal to 20:80. In addition to the interpretation of findings provided by authors (increased responsiveness to false feedback and decreased reinforcement sensitivity), the reversal learning impairment may be explained by decreased sensitivity to changes in probability of reward associated with each option before and after reversal, and a less steep devaluation of response option with increases in uncertainty cost.

In the present investigation by contrast there is no probability component (i.e., the relationship between stimulus and outcome is deterministic); the only way in which the stimulus is devalued is by the rats' repeated experience with omission of reward delivery. Whereas, risk discounting could play a role in the former study, it is not a factor in the present investigation.

Thus, the present findings provide novel evidence for the lack of a role for 5-HT in BLA in deterministic reversal learning. However, it needs to be noted that although the methods implemented in the present investigation are similar to ones previously reported, there are several important distinctions which might have masked the effect of treatment on behavior. One of the important distinctions is animals' experience with *both* the discrimination and reversal tasks prior to lesion. Rats already acquired the knowledge of the optimal strategy to learn the task. In Izquierdo et al. (2013), where potentiated responses to negative feedback and enhanced reversal learning performance following lesions were observed, the animals were only pre-trained before the surgeries and both of the tasks were introduced only following the recovery period. In Rygula et al. (2014) monkeys had a preoperative experience with discrimination but not reversal learning. This observation is particularly interesting as it suggests that the behavioral alterations observed in the previous investigations could have resulted from changes in strategy learning. Early studies in monkeys with amygdala lesions conducted by Schwartzbaum and Poulos (1965) showing an impairment in the transfer of learning only during initial reversals in a set, provide support for this interpretation.

BLA is critically important in detecting and updating response strategy following a change in reward-predicting rules or cues (Coleman-Mesches et al., 1996; Baxter and Murray, 2002; Liao and Chuang, 2003; Belova et al., 2008; Ostrander et al., 2011). Conflict detection is particularly important during the first experience with the reversal learning. Rygula et al. (2014) observed that the impairment on reversal learning resulted from increased responsiveness to misleading feedback and decreased overall reinforcer sensitivity, which suggests a decreased ability to integrate reward information across time. In a probabilistic learning task it is likely to manifest in an increased win-stay/lose-shift strategy, regardless of feedback veracity and lead to impaired responses after misleading feedback. Thus, in the present experiment, wherein the first experience with the reversal occurred with intact BLA, the demand of strategy learning is reduced, and the retention of the already-learned task rules may be sufficient to guide responses. Future studies need to address this question by systematically implementing different timelines of lesion or inactivation. 5-HT depletions of BLA may impair reversal acquisition only if administered after surgery, without preoperative training.

Another interesting possibility arises from the consideration of the specificity of depletions. Previous studies reporting correlations between 5-HT levels and reversal learning performance have considered global depletions, which in addition to BLA also produced significant 5-HT reductions in OFC, mPFC, and hippocampus among the brain regions examined (Masaki et al., 2006; Izquierdo et al., 2012). It is therefore plausible that the negative correlations between the reversal learning performance and 5-HT levels were driven by neurotransmitter concentrations in other brain regions, not amygdala. Future research may benefit from direct manipulation of 5-HT neurotransmission in PFC or hippocampus of animals performing a reversal learning task to understand the region-specific contribution of 5-HT in the previously reported impairment. Another possibility is that compromised 5-HT signaling in one brain region may be insufficient to produce an appreciable behavioral impairment. Instead, systems manipulations with depletions targeted in several interconnected brain regions might be necessary. Though the 5-HT depletions were restricted to BLA in the present study, this method precludes differentiation of 5-HT receptor subtype involvement in reward learning, an avenue of inquiry perhaps best pursued with more specific pharmacology or chemogenetic targeting.

## Author contributions

JO and AI designed the research; JO, AS, AK, EH, and AB performed research; JO, AS, and AI analyzed data; JO, AS, and AI wrote the paper.

### Conflict of interest statement

The authors declare that the research was conducted in the absence of any commercial or financial relationships that could be construed as a potential conflict of interest.
